# Implementation of practices shapes the effectiveness of agricultural diversification for arthropod related ecosystem services: a meta-analysis

**DOI:** 10.1007/s13593-025-01082-7

**Published:** 2026-02-26

**Authors:** Gaëtan Seimandi-Corda, Chloe MacLaren, Kevin Tougeron, Johannes Forkman, Jess Hood, Andrew Mead, Amelia Dixon, Samantha M. Cook

**Affiliations:** 1 Rothamsted Research, Harpenden, Hertfordshire UK; 2 UMR AGIR, Université de Toulouse, INRAE, Castanet-Tolosan, F-31326 France; 3 International Maize & Wheat Improvement Centre (CIMMYT), Southern Africa Regional Office (SARO), P.O. Box MP163, Harare, Zimbabwe; 4 Department of Crop Production Ecology, Swedish University of Agricultural Sciences, Uppsala, 75007 Sweden; 5 Ecology of Interactions and Global Change Laboratory, Institute for Biosciences, Université de Mons, Mons, Belgium

**Keywords:** Plant diversity, Pests, Flower margin, Intercropping, Insect

## Abstract

Agricultural intensification has increased food production but has also caused significant environmental degradation and biodiversity loss. Diversification practices can mitigate these impacts while sustaining yields. While previous meta-analyses have examined their effects on arthropod populations and associated ecosystem services, focusing on individual practices or treating them as a whole, this is the first study to compare a wide range of diversification strategies across specific arthropod groups and assess how management factors, such as plant diversity, spatial configuration, and sowing time, modulate their outcome. Clarifying these mechanisms is crucial for optimizing diversification practices and enhancing their adoption by farmers. We conducted the most up-to-date and comprehensive meta-analysis to evaluate the effects of four diversification practices (agroforestry, intercropping, flower resource addition, and maintaining semi-natural habitats) on arthropods (parasitoids, predators, pollinators, and herbivores), their associated ecosystem services (predation, parasitism, pollination), and disservices (herbivory), using 19,421 data points from 449 publications. On average, all diversification practices increased beneficial arthropod populations and their services by 41%, compared to a non-diverse control, and reduced herbivore abundance and plant damage by 33%. Intercropping was particularly effective, reducing herbivore abundance and damage by 39% and 30%, respectively, while increasing predator and parasitoid populations by 48% and 56%. Other practices showed no consistent effects, likely due to high variability across studies. For the first time, we tested the influence of management factors and found complex effects on parasitoid abundance and parasitism, highlighting the need for context-specific approaches. These results highlight the importance of tailored policies and targeted research to support the adoption of diversification practices. Effective implementation can reduce reliance on insecticides while simultaneously promoting ecosystem services and supporting the transition to sustainable agricultural systems.

**Supplementary information:**

The online version contains supplementary material available at 10.1007/s13593-025-01082-7.

## Introduction

Agricultural intensification, achieved through increasing mechanization, high inputs of fertilizers and pesticides, expanding field sizes, and reducing the number of crops grown, has resulted in a remarkable surge in food production (Grassini et al. 2013). However, agricultural intensification is also recognized as a primary driver of global environmental changes, exerting substantial adverse impacts on biodiversity (Raven and Wagner 2021). These biodiversity impacts, in turn, affect the provision of essential ecosystem services required to support agricultural production, including pest regulation and crop pollination (Ratnadass et al. 2012; Emmerson et al. 2016; Kass 2020), thereby posing a global threat to food production. Consequently, it is imperative to develop sustainable farming practices that can sustain yields while minimizing impact on the environment.

In recent years, agricultural diversification, especially practices where multiple plant species are grown simultaneously, has been advocated to reduce the negative impact of farming practices on biodiversity while maintaining high productivity (Kremen et al. 2012; Isbell et al. 2017; Brooker et al. 2023; Bommarco 2024). Diversification can encompass an increase in crop diversity temporally through longer and more varied crop rotations, as well as spatially via intercropping, or an increase in non-crop plant diversity through the addition of flower margins, agroforestry, or the maintenance of semi-natural habitats (SNH), such as hedgerows proximate to crops (Kremen et al. 2012; Hufnagel et al. 2020; Wan et al. 2020). Global syntheses have shown that plant diversification practices increase biodiversity compared to highly simplified cropping systems (Lichtenberg et al. 2017; Dainese et al. 2019), and also that they can have positive effects on water and soil quality, while reducing greenhouse gas emissions (Tamburini et al. 2020; Beillouin et al. 2021). Moreover, diversification practices have exhibited beneficial impacts on ecosystem services essential to agriculture, including pest and disease management, and the promotion of insect pollinators (Wan et al. 2020; Zamorano et al. 2020).

Ecosystem services related to the populations and activities of insects and other arthropods are critical to support agricultural production. On one hand, insect pests cause approximately 18% of global yield losses (Oerke 2006), while on the other hand, insect pollinators contribute to around 8% of food production (Aizen et al. 2009). Crop diversification can affect arthropods via both top-down and bottom-up effects. Bottom-up effects occur when plant diversity has a direct effect on herbivores, altering their populations or the amount of damage they cause to crops. Such effects can be observed when additional plant species disturb host plant location, visually hide the crop, or have a repellent or attractive effect on herbivores (Ratnadass et al. 2012). Top-down effects occur when plant diversity supports biocontrol agents (i.e., predators and parasitoids) by providing resources such as pollen, nectar, alternative hosts and prey, or habitat and refugia (Ratnadass et al. 2012). These resources support the population of beneficial insects that suppress herbivore numbers, thereby ultimately boosting crop yield or reducing the need for pesticides to sustain yields. The addition of plant species providing floral resources will also support pollinator populations and enhance the yield of some crops (Nicholls and Altieri 2013).

Previous syntheses that considered all plant diversification practices together have shown a trend of positive effects on beneficial insects (predators, parasitoids, and pollinators) and their associated services, as well as a decrease in herbivore abundance and crop damage (Lichtenberg et al. 2017; Wan et al. 2020, 2022; Tamburini et al. 2020). Some syntheses have focused on specific practices such as intercropping (Iverson et al. 2014; Rakotomalala et al. 2023), agroforestry (Pumariño et al. 2015; Perez-Alvarez et al. 2023), or the addition of flower resources or SNH (Albrecht et al. 2020; Crowther et al. 2023; Jachowicz and Sigsgaard 2025) and have generally reached similar conclusions. These studies give a generally positive view of diversification practices, yet their implementation by farmers is still limited. Reasons behind the barriers for adoptions of diversification practices are multiple and complex involving economic, sociological, and psychological factors but also limitations in the availability of specific knowledge about the effect of diversification practices (Blesh et al. 2023). Previous studies have not compared the effects of different diversification practices. A few have compared specific practices, for example, SNH with flower resource addition (Albrecht et al. 2020), or intercropping, agroforestry, and the presence of hedgerows (Beillouin et al. 2021; Jaworski et al. 2023). However, these studies focused on a narrow range of responses such as pest regulation and pollination services (Albrecht et al. 2020), or pest (including pathogen) regulation only (Beillouin et al. 2021; Jaworski et al. 2023). They did not distinguish between the abundance of the arthropods and the services they support, nor between effects on different functional groups such as parasitoids and predators. A comprehensive evaluation of the effects of diversification on the arthropod community as a whole, and the services and disservices provided, is therefore still lacking.

Moreover, the manner in which diversification practices are implemented and managed by farmers, including factors such as the number of added plant species and their spatial configuration, has often been overlooked. Only Albrecht et al. (2020) and Jachowicz and Sigsgaard (2025) examined the effect of the number of flower species. The first study also assessed how the age of the flower margin affected the services they studied. Rakotomalala et al. (2023) investigated the effect of different configurations of intercropping or combinations of plant families on pests and beneficials. The three studies showed that management factors are key moderators of the outcome of diversification practices. Understanding the effect of these management factors is crucial to provide specific knowledge to farmers and enable them to identify the most efficient practices and elucidate why certain farming systems yield better outcomes compared to others.

In this study, we focus on four farm- and landscape-level diversification practices: (1) intercropping, i.e., the practice of growing two or more crops in the same field at the same time; (2) agroforestry, where woody perennials and crops are grown on the same land; (3) the addition of flower resources, typically achieved through sown, non-woody flower strips or margins surrounding or within crops; (4) the presence of SNH, such as grasslands, woodlands, and hedgerows, adjacent to the crop (Table S1). We reviewed articles published between 1980 and 2024 and collected 19,421 data points from 449 publications. Through meta-analysis, we aimed to take a closer look at differences in the effects of the four diversification practices on different groups of arthropods (herbivores, predators, parasitoids, and pollinators), the services they support (predation, parasitism, and pollination), and their impact on the crop (crop damage via herbivory).

To address the critical knowledge gap regarding how best to implement diversification, we also tested the effect of key variables commonly used across studies to describe the management of each diversification practice. These included (i) for intercropping: the number of intercropped species, the type of associated crops (cash crops or companions), sowing timings, and spatial configurations; (ii) for flower resources addition, the effect of flower species richness, patch size, duration, and proximity to the crop; (iii) for SNH, habitat type (hedgerows, woodland, grassland, grassy strips) and distance from the sampling location; (iv) for agroforestry systems, layout type (alley cropping and managed forest) and product (fruits and nuts, timber, no specific purpose).

## Materials and methods

### Data collection

**Fig. 1:**
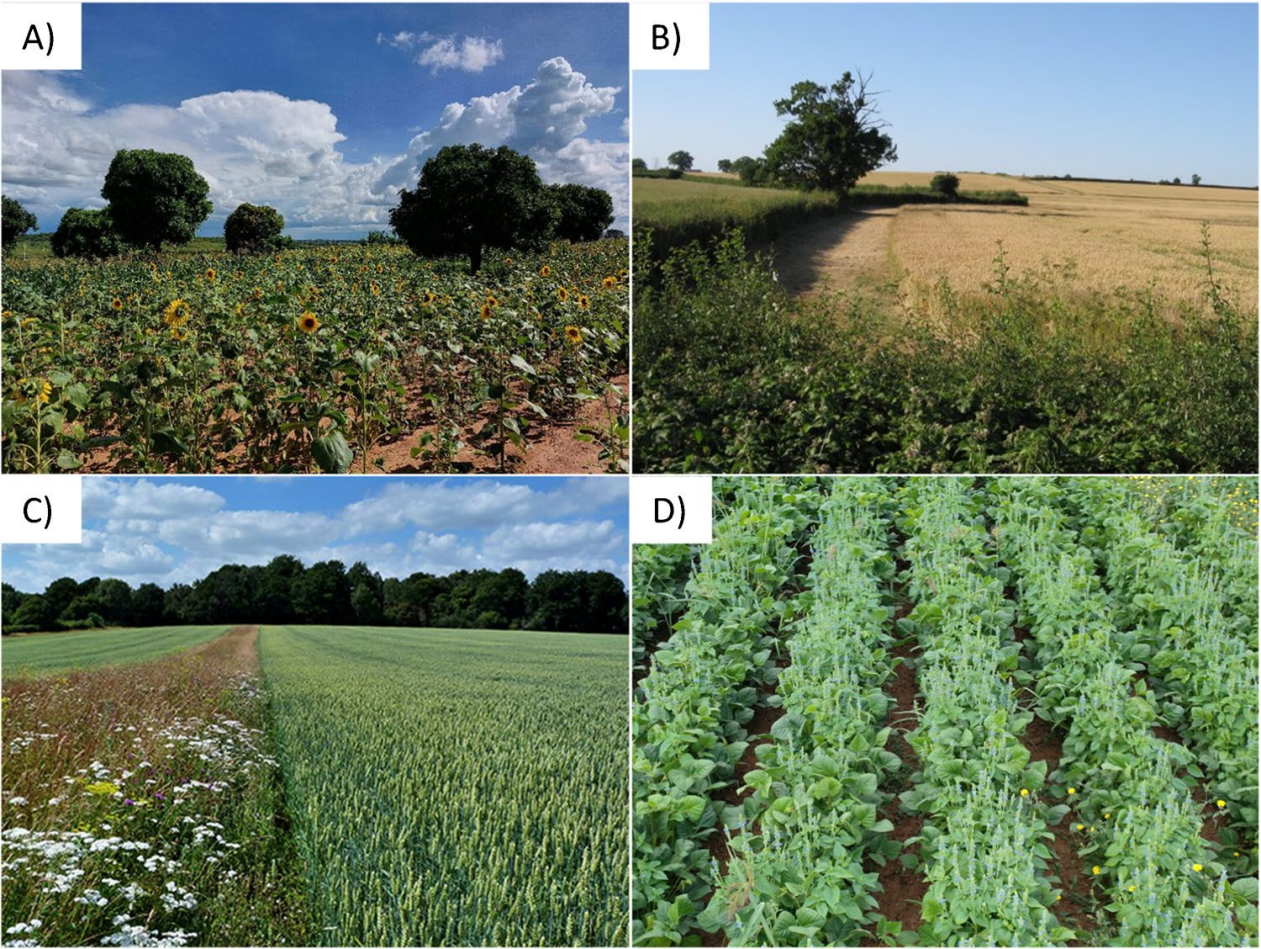
Crop diversification practices considered in the analysis: **A** agroforestry (mango trees and sunflower), **B** maintenance of semi-natural habitats (hedgerow), **C** flower addition (flower strip along a cereal field), **D** intercropping (chia and mungo beans). Photocredit: Chloe MacLaren (**A**), Doug Lee (**B**), Sarah Hulmes (**C**), Fanny Raoux (**D**).

Our search targeted articles presenting data collected in a context of agricultural plant diversification on the abundance of arthropods (herbivores, predators, parasitoids, pollinators), and/or on the provision of services (predation, parasitism, pollination) and disservices (herbivory) performed by arthropods. The diversification practices we considered were agroforestry, the addition of flower resources, intercropping, and the maintenance of SNH proximate to the crop (Fig. 1). The search was conducted using the Web of Science database (Core Collection), with the search string: (undersow* OR underseed* OR interseed* OR intercrop* OR “companion crop*” OR “companion plant*” OR “living mulch” OR interplant* OR “mixed crop” OR “flower strip*” OR “wildflower strip*” OR “flower margin” OR “field margin” OR “flower border” OR agroforestry OR “alley cropping” OR “trap crop*” OR hedgerow OR “field edge” OR “field boundaries” OR “crop diversification”) AND (insect OR pollinat* OR herbivor* OR predat* OR parasit* OR biocontrol OR “biological control” OR “pest control” OR “natural enem*”). Were removed from the search the terms: nematod* OR pathogen* OR slug*. It was undertaken for the first time on 7/01/20, and then updated regularly up to 11/06/24 to account for recently published articles (see Supporting information for details of the search) and accounted for studies published since 1980. In total, 3447 articles were identified using Web of Science.

Each paper was then carefully inspected, and data extracted if they reached the following six criteria: (1) The article deals with one of the diversification practices considered. (2) The article presents both high (treatment) and low (control) levels of plant diversity. Low diversity treatments were usually a monoculture (a single crop species grown on a plot) or a grassy margin. Studies comparing multiple flowering margins without a control that did not include spontaneous vegetation, grassy margins, or a crop were considered to lack a low-level plant diversity control and were discarded. However, if a low diversity control was present, we included articles with multiple different high diversity treatments (which were grouped or separated depending on the analysis, see Section 2.2). (3) The article deals with arthropods (insects, arachnids, etc.). (4) The article or data available in Supporting Information presents data that can be extracted from text, tables, or figures. Reviews and meta-analyses were not considered. Articles only presenting results of inferential statistics, such as *p*-values, associated with tests between different diversification levels were not considered. (5) The article presents data from samplings conducted in the crop. Studies reporting, for example, only the abundance of arthropods in different SNH were not considered. (6) The article presents data from “agriculturally realistic” experiments. Data were not considered if the experiment was done on potted plants (except if this was a standard agricultural practice, for example, in horticulture), or if the experiment was done in controlled conditions, e.g., a laboratory or experimental glasshouses.

A total of 427 articles met these criteria and were analyzed carefully to extract data (see Fig. S1 for details of the reason for excluding articles). This dataset was supplemented with 22 articles obtained from the reference lists of other published meta-analyses conducted on agricultural diversification (Letourneau et al. 2011; Lichtenberg et al. 2017; Albrecht et al. 2020; Wan et al. 2020; Zamorano et al. 2020). Each article was then inspected and data extracted from text, figures, tables, and Supporting Information. Criteria were then applied to remove redundant values belonging to the same article (see Supporting Information). For each observation, the diversification practice used as well as information to characterize how the practice was implemented (e.g., management factors) were also recorded (see Table S1).

### Statistical analysis

**Fig. 2:**
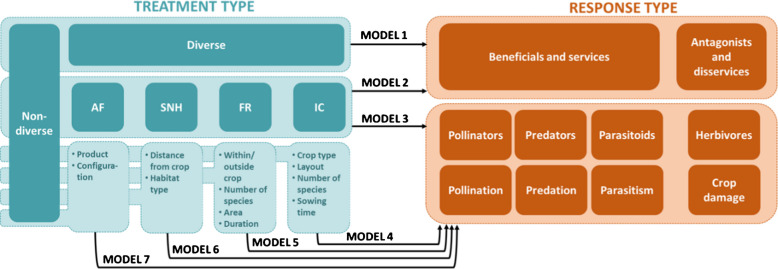
Explanatory variables and levels used in models tested in a meta-analysis to examine the effect of different levels of plant diversification practices (agroforestry (AF), semi-natural habitat (SNH), flower resource addition (FR), intercropping (IC)) on beneficial (pollinators, predators, parasitoids) and antagonistic (herbivorous) arthropods and associated ecosystem services (pollination, predation, parasitism) or disservices (crop damage). For Models 1–3, light shaded boxes represent variables used in the models, with dark shaded boxes indicating the levels of these variables. Levels placed below each other are nested with higher levels. “Non-diverse” was always the control level to which all other levels of plant diversification treatments were compared. For Models 4–7, the light shaded boxes for “treatment type” indicate each separate model, with text listing variables included in each model (levels not shown, see Table S1). Both the “treatment type” and “response type” variables are explanatory variables in each model, while the response variable always comprised the observations collected on arthropod abundances and service provision from the articles.

The data extracted from the articles comprised observations of the arthropod response (abundance of arthropods or provision of services/disservices) to different treatment types (non-diverse control and different diversification practices), alongside information about the response type (what kind of arthropods or services; Fig. 2). Our analysis aimed to determine how the response differed depending on both the treatment type and the response type; in other words, testing how different types of arthropods and services responded to different diversification practices. We took a hierarchical approach, partly inspired by Wan et al. (2020), with initial models providing a general overview of positive and negative effects of diversification, and subsequent models investigating associations between specific categories of diversification practice, arthropods, and services. First, we evaluated whether diversification in general (all four practices grouped together) had an overall effect on two response groups: beneficial arthropods and ecosystem services (including predators, parasitoids, pollinators, predation, parasitism, and pollination) and antagonistic arthropods and ecosystem disservices (including herbivores and crop damage) (Model 1). Second, we tested whether the four diversification practices differed in their effects on beneficial arthropods and ecosystem services and on antagonists and disservices (Model 2). The third step was to test whether the diversification practices had different effects on different groups of arthropods and services/disservices (Model 3). Models 1–3 ran on our full dataset of 19,421 observations from 449 papers. Subsequent models tested the effect of different methods of implementing each diversification practice (Table S1) on the abundance of different arthropod groups and the provision of services/disservices (Models 4–7). For example, Model 4 compares control treatments without intercropping to intercrop treatments with different numbers of species, which were sown at different times, or in different layouts (Fig. 2, Table S1). Given that the implementation variables are nested within the relevant diversification practice, each of these models was run on the subset of data pertaining to each diversification practice.

For each model, we used the arm-based network meta-analysis method presented by Piepho et al. (2012, 2024). Arm-based meta-analysis involves extracting response data from each study for all treatments tested within that article (including the control), and then using a model to estimate the mean response for each treatment across all articles. Post hoc comparisons were then used to assess overall differences between each treatment and the control, and differences within response categories (e.g., in Model 1, comparisons were made between “non-diverse” and “diverse” within “beneficials and services” and within “antagonists and disservices”). This method differs from the traditional contrast-based meta-analysis method, where the differences between the treatments and the control are first calculated within each article (e.g., the log response ratio) and a model is used to estimate the mean difference across articles. The arm-based method is preferable for meta-analyses containing studies where the effects of multiple treatments are compared to a common control, because the method provides a better adjustment for within-study correlations between treatments, making it easier to split direct from indirect evidence (Piepho et al. 2024).

The response variable used in all our models was the log of the response recorded for each observation in each article. Taking the log allowed us to firstly correct for an increasing variability in the response as the mean response increased, and secondly to group together articles where the response was measured in different units and on different scales. The difference between two log-transformed values is the same as the log of their ratio, meaning that differences between treatment means calculated on the log scale could be back-transformed to ratios, removing the units.

We implemented arm-based meta-analysis in R version 4.4.2 (R Core Team 2024) using the packages *glmmTMB* (Brooks et al. 2017) and *emmeans* (Lenth and Lenth 2018). The meta-analysis models were mixed effects linear models (which assumed a normal distribution and homoscedasticity of the residuals, after log transformation of the response). The treatment type and response type factors (Fig. 2), their interaction term, and the article identity were included as fixed effects. Interaction terms between each factor and article identity were included as random effects. The models were constructed using the *glmmTMB()* function, which enabled us to appropriately partition within vs between study variance by setting the “dispformula” argument to “~0 + obs,” where “obs” was a unique (categorical) identifier for every individual observation. The “start” and “map” arguments were used to set the starting value for each observation to the log of the inverse of the number of replicates that observation was based on. In meta-analysis, it is traditional to use the variance calculated by squaring the standard error (SE) for the observation extracted from the article. However, SEs can be calculated in a variety of ways across different studies, depending on the statistical techniques used. Furthermore, they are not always reported, and missing SEs have to be estimated. These differences in SE calculation methods can introduce bias into meta-analysis models, resulting in inaccurate mean estimates (regardless of whether they are arm-based or contrast-based). We found this to be a problem with our dataset and thus replaced the variance with the inverse of the number of replicates, which reduces the weighting given to observations derived from a small number of replicates. Where an article did not report the number of replicates, we conservatively assumed that the observation was derived from only one replicate.

Differences between treatments were assessed using the “~ctrl.vs.trt” method within the *contrasts()* function of *emmeans* to provide estimated treatment ratios (obtained by back-transforming differences between the log treatment means). The degrees of freedom were not correctly calculated for this type of model by *emmeans* (at the time of writing, it appeared the Kenward-Roger or Satterthwaite methods are not available for *glmmTMB* models in *emmeans*), so we manually adjusted these to the number of observations minus the number of fixed-effects parameters. Confidence intervals (95%) for the treatment ratios were also calculated by *emmeans*, which we used to determine whether the estimated ratio was significantly different from 1 (a ratio of 1 = no difference).

To assess potential publication bias in the dataset, we generated funnel plots by plotting precision (1/√number of replicates) against the effect size. Since the arm-based meta-analysis does not directly rely on effect size calculations, we computed an effect size (log response ratio) for this purpose. For each study, this effect size was obtained by averaging the results of non-diversified and diversified treatments, considering the type of diversification practice, the classification of arthropods (beneficial vs antagonistic), their associated service or disservice, and the number of replicates. Separate funnel plots were then generated for each diversification practice and for each arthropod category (beneficial/service or antagonistic/disservice), and these plots were visually inspected to identify potential asymmetries indicative of publication bias.

## Results and discussion

### Studies published represent a good geographical coverage but are unequally distributed

The meta-analysis includes 19,421 observations (12,035 increased plant diversity treatments and 7386 non-diverse controls) from 449 studies. Intercropping comprises the majority of the diversified treatments (9234 observations, 289 studies), followed by flower resource addition (1821 observations, 106 studies), semi-natural habitat (SNH) (511 observations, 36 studies), and agroforestry (469 observations and 28 studies). In terms of response variables, most observations were on herbivore abundance (9721 observations from 302 studies), followed by predator abundance (4839 observations from 213 studies), parasitism rate (1523 observations from 88 studies), crop damage by herbivores (1408 observations from 88 studies), parasitoid abundance (1109 observations from 71 studies), pollinator abundance (347 observations from 44 studies), predation rate (421 observations from 37 studies), and finally pollination (53 observations from 14 studies).

**Fig. 3:**
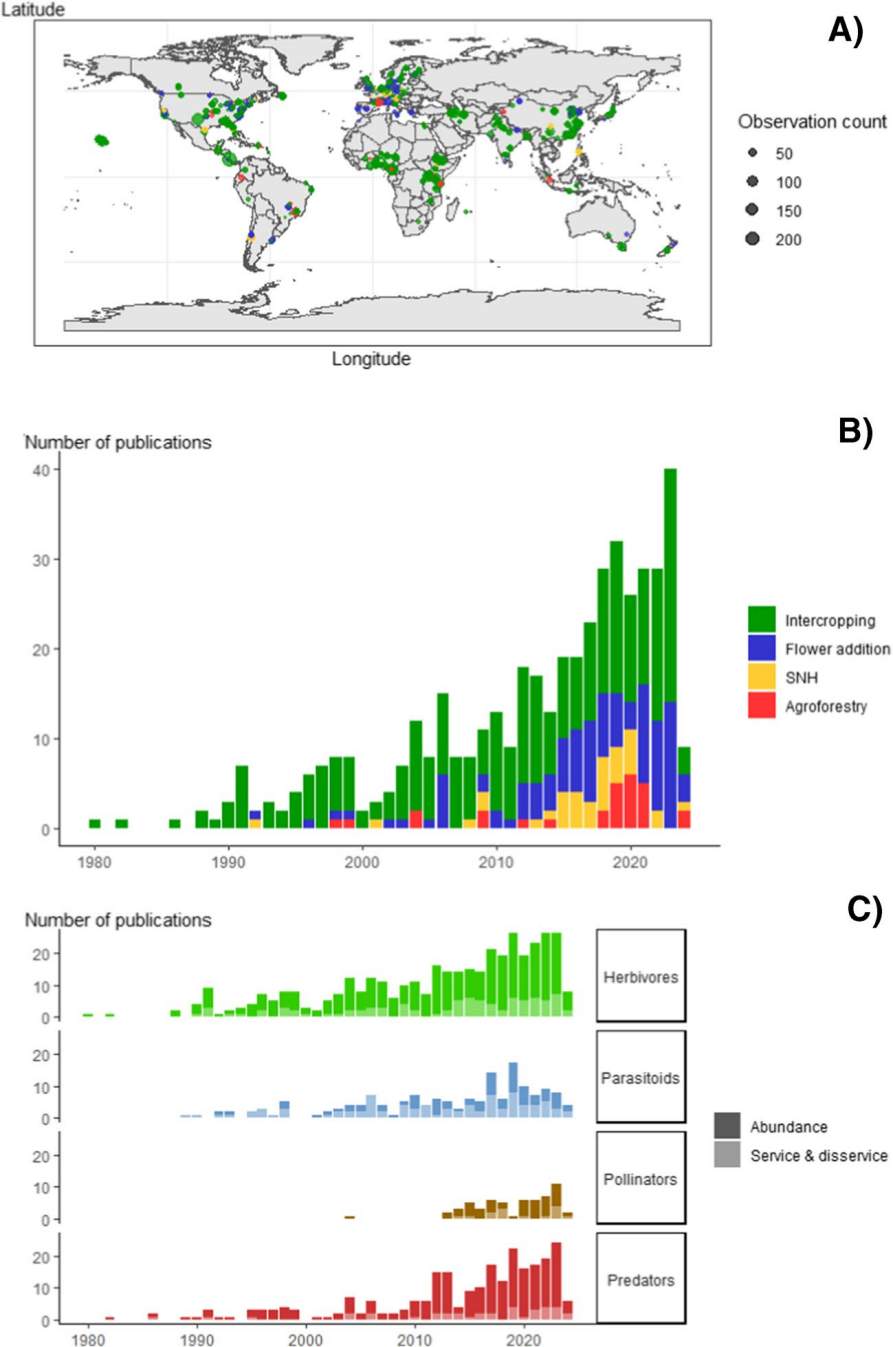
Map and temporal trend in the publication of the data used for the meta-analysis. **A** Map of the location of the different observations collected in the meta-analysis on the impact of plant diversification practices on arthropod abundance and ecosystem services. Duplicated locations were removed, and some observations could not be located because of a lack of information in the articles. **B** Number of publications used in the meta-analysis published per year between January 1980 and June 2024. Dots and bar colors represent different diversification practices: green, intercropping; blue, flower resource addition; yellow, semi-natural habitat (SNH); and red, agroforestry. **C** Number of publications used in the meta-analysis published per year, split between arthropod functional groups (herbivores, parasitoids, pollinators, and predators), with dark shade representing the abundance of arthropods and light shade the ecosystem services or disservices they cause.

Studies used for the analysis were published between January 1980 and June 2024, peaking in 2023 with 38 publications (Fig. 3B). For most response categories, studies started to be published on a frequent basis around the 1990 s and increased over time, except for pollinators and pollination, where studies started to be published frequently only after the 2010 s (Fig. 3C).

The included studies encompass all continents with cropped land (Fig. 3A): Europe (3235 observations from 129 studies), North America (4538 observations from 110 studies), Asia (5445 observations from 94 studies), Africa (3619 observations from 66 studies), South America (1683 observations from 33 studies), and Oceania (904 observations from 20). Intercropping is well represented in all continents, with a minimum of 12 studies found in Oceania and a maximum of 80 in Asia. However, the others are mainly found in Europe or North America, and in particular, SNH is not represented in Africa or Oceania. This unbalanced geographic distribution of studies reflects past and current discrepancies in research conducted in different countries.

Funnel plots created to check for publication bias do not show major signs of publication bias for the different diversification practices and the classification of arthropods (beneficial vs antagonistic) and their associated service or disservice (Fig. S4).

### Plant diversification has positive effects on ecosystem services and pest management

**Fig. 4:**
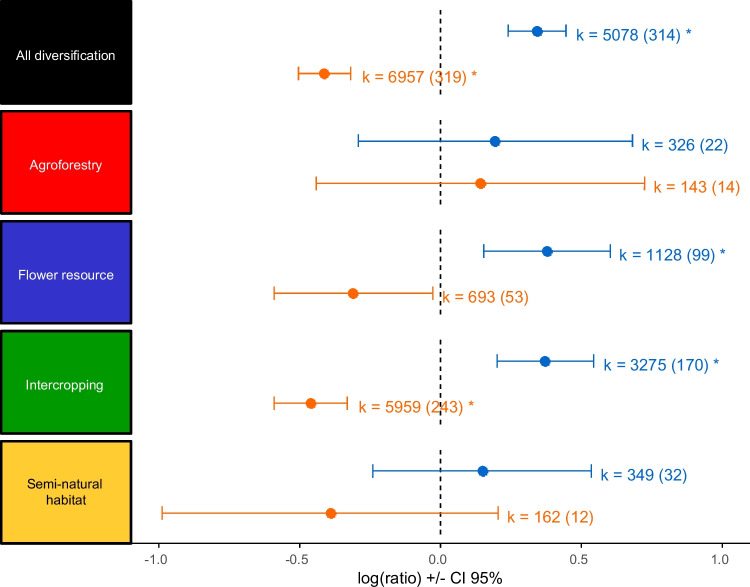
Log of ratio (± CI 95%) of the effect of plant diversification practices on beneficial arthropods and ecosystem services (blue) and antagonists and disservices (orange) for all diversification practices and individual practices. *k* represents the number of observations in diversified treatments, and the numeral in brackets represents the number of publications per practice and response categories. Asterisk (*) highlights significant differences between control and diversified treatments.

When comparing all diversification practices to non-diverse control practices, we observed an overall positive effect of diversification on beneficial arthropods (parasitoids, predators, and pollinators) and the ecosystem services they provide, leading to an average 41% (CI 95% 27–56%) increase, while reducing herbivore abundance and damage by an average of −33% (CI 95% −39 to −26%) (Fig. 4, Table S2). These results align with previous meta-analyses highlighting the positive effect of plant diversification practices, including agroforestry, addition of flower resources, intercropping, and maintenance of SNH (Lichtenberg et al. 2017; Wan et al. 2020, 2022; Tamburini et al. 2020; Beillouin et al. 2021). The observed benefit can be attributed to a combination of bottom-up and top-down mechanisms. Addition of flower resources, maintenance of SNH, and agroforestry are often implemented to increase the population of natural enemies in the crop, leading to higher ecosystem services that consequently negatively affect arthropod pest abundance and their damage to crops (Ratnadass et al. 2012). Intercropping can also lead to increased natural enemy populations by providing them with resources including nectar, pollen, and secondary prey. Intercropping is commonly implemented with mixtures of cereals and legumes (Kirsch et al. 2023) where legume flowers or extrafloral nectar can be used by some natural enemies for food. These species mixtures often also host populations of aphids used by predators as an alternative prey or a source of honeydew (Luquet et al. 2021). A direct effect of plant diversity on herbivores can also occur in intercropping, where practices such as trap-cropping and repellent-cropping disrupt herbivore colonization of crop plants (Shelton and Badenes-Perez 2006).

The effect of plant diversification on arthropods and their related ecosystem services is highly variable between studies, so although the overall trend is positive, a lot of data are needed to reliably detect a significant positive mean effect. This variability between studies can be observed in our results: the means for treatment and/or response categories with fewer observations from fewer studies have wider confidence intervals. This variability justifies the need to explore why diversification is more effective in some cases and less in others. In our subsequent analyses, we explore whether different arthropod groups and services respond differently to each diversification practice and whether the implementation and management of each diversification practice matter. 

### Diversification practices have different effects on arthropods and their services

Few meta-analyses have specifically compared how different diversification practices affect arthropod populations and the ecosystem services they provide to crops. Letourneau et al. (2011) distinguished between intercropping and flower addition, finding that both practices enhanced services to crops while reducing herbivore pressure. Similarly, Albrecht et al. (2020) compared the effects of SNH and flower addition, showing a significant increase in pest control (predation and parasitism) with the addition of flowers alone. Our results concur with these findings: significant differences between monocrops and diversified plots are observed only for the addition of flower resources and the use of intercropping (Fig. 4, Table S2). These two practices lead to increases in the abundance of beneficial arthropod populations and their associated ecosystem services, and in the case of intercropping, to a reduction in herbivore abundance and crop damage. Intercropping and the addition of flower resources are the two practices for which we have the most data (Fig. S2), which might explain why the effects we observed are clearer than for other practices.

Beillouin et al. (2021) further divided diversification practices, including agroforestry, intercropping, cultivar mixtures, cover crops, and rotations, and demonstrated that both intercropping and agroforestry enhance pest and disease control. We did not find a positive result for agroforestry, but we have data from relatively few agroforestry studies (28) compared to intercropping (289) and flower resources (106), reducing the potential to detect a significant effect in the context of high between-study variability. Semi-natural habitats (with 35 studies) also do not yield statistically significant differences compared to monocrops, but follow a similar trend to intercropping and flower resources in a reduction of antagonists and an increase in beneficials (Fig. 4).

**Fig. 5:**
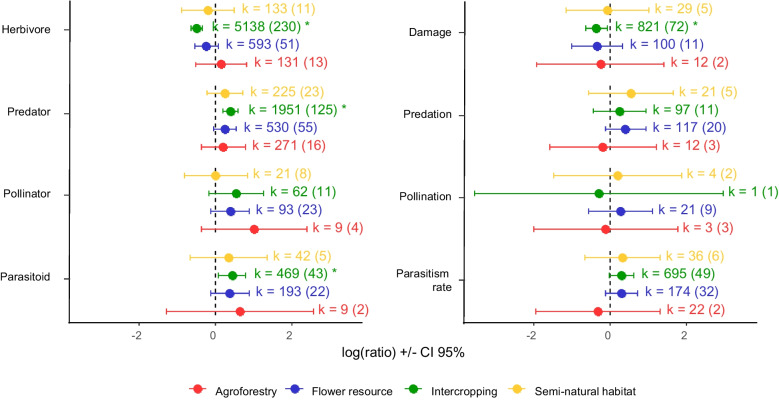
Log of ratio (± CI 95%) of the effect for different response categories and different cropping practices. Green, intercropping; blue, flower resource addition; yellow, SNH; and red, agroforestry. Asterisk (*) highlights significant differences between control and diversified treatments. *k* represents the number of observations in diversified treatments and in brackets the number of publications per practice and response categories.

When examining the response of specific types of arthropods and specific services/disservices, significant effects were observed only when intercropping was implemented (Fig. 5, Table S2). Intercropping leads to a reduction of herbivore abundance by 39% (CI 95% 47–29%) compared to monocropping, a reduction of plant damage (–30% on average (CI 95% −46 to −8%)), and an increase in the abundance of predators and parasitoids on average of 48% (CI 95% 21–82%), and 56% (CI 95% 9–122%), respectively (Fig. 5). No significant effects of plant diversification were detected for predation, parasitism, pollinator abundance, or pollination services. However, despite the lack of statistical significance, general trends for most of the practices indicate an increase in beneficial arthropod populations and ecosystem services, coupled with a reduction in herbivore populations and plant damage. Given the limited data availability for these categories, we are hesitant to conclude that the lack of significance indicates no effect across all categories; rather, it seems more plausible that there are small positive effects that are obscured by the high variability in outcomes between studies.

Other syntheses have also tested some of the categories shown in Fig. 5, finding similar results to ours. Specifically, Letourneau et al. (2011) also observed a reduction in herbivore abundance in intercropping systems, but not when flower margins are used. Albrecht et al. (2020) also failed to observe an effect of SNH and flower resource addition on pollination, but did observe a positive effect of flower addition on natural enemy abundance and parasitism rate. The latter is also hinted at in our data; the difference may relate either to the type of analyses used (Albrecht et al. 2020 were able to work with the raw data and thus account for more variability) or to the number of studies used (14 out of the 27 studies used in Albrecht et al. 2020 are common with the 106 studies included for SNH and flower addition in our analysis).

### The effect of management of diversification practices varies among different arthropods

**Fig. 6:**
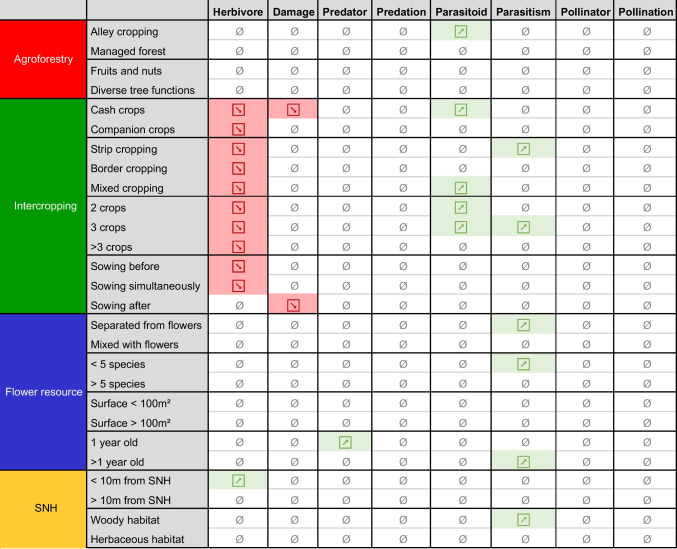
Summary of the effect of different management variables in studies using different plant diversification practices compared to monocrops on different response categories. Red arrows indicate a decrease of the response for a level of the variable tested, a green arrow indicates an increase, and a barred zero indicates that not enough points were available to test this effect. Colored cells (red or green) show significant results. Explanations of each category are provided in Table S1, while details of the number of observations and studies for each level of the variables are presented in Table S3.

In our investigation of the effects of management factors related to each diversification practice, we found significant but contrasting effects on herbivore abundance compared to parasitoid abundance and parasitism rate (Fig. 6, Table S2). Intercropping consistently suppressed herbivore abundance, while neither addition of flower resources nor SNH affected herbivores, regardless of how these diversification practices were managed. In contrast, parasitoids and parasitism were affected by management, with specific intercropping, flower resource, and SNH strategies leading to increased parasitism, and certain intercropping strategies leading to increased parasitoid abundances.

The link between the abundance and herbivore damage or parasitism was not clear. The general direction of effects between arthropods and services was consistent: intercropping reduced both herbivore abundance and damage, while usually increasing both parasitoids and parasitism. However, a significant effect on arthropod abundances was not usually associated with a significant effect on the associated service, and vice versa. This lack of relationship can be explained by the fact that other aspects of arthropod communities can impact the provision of services. Trait composition of communities can mediate this relationship, with examples of predator communities with smaller community weighted mean body length being more effective at providing predation on aphids than larger ones (Rusch et al. 2015). Moreover, the total abundance of ecosystem service providers can hide interactions within this community, with intra-guild predation or competition, typically between predators and parasitoids, having potential negative effects on the delivery of the service (Frago 2016). Although almost all intercropping strategies significantly reduced herbivore abundance, crop damage was reduced under only two intercropping strategies. Similarly, parasitism rates were enhanced in several management categories of intercropping, addition of flower resources, and SNH that were not also found to enhance parasitoid abundance. This suggests that different factors may be important in regulating arthropod populations and their activity, so that one cannot simply assume that more or less of a given arthropod type will equate to higher or lower provision of their associated (dis-)service.

#### Intercropping

In intercropping, previous studies have shown that the spatial arrangement of crops can affect arthropods (Rakotomalala et al. 2023). Closer spacing between plant species can enhance the proximity of natural enemies to herbivores while simultaneously complicating herbivore host-plant location. Border cropping, a common strategy for trap-crops, involves sowing species that are more attractive to herbivores than the crop along crop edges to divert pests from the main crop (Shelton and Badenes-Perez 2006). This approach is expected to decrease herbivore abundance without significantly affecting natural enemies or pollinators. Our findings partially align with these predictions: increased parasitoid populations and parasitism rates were observed in configurations where crops were closely interspersed (e.g., strip and mixed cropping) but not in border cropping (Fig. 6, Fig. S5). Strong evidence supports a reduction in herbivore populations across all tested intercropping configurations, highlighting the effectiveness of this practice in decreasing herbivore pressure (Fig. 6, Fig. S5).

While a positive relationship between plant species diversity and ecosystem services is well-documented in natural systems (Wan et al. 2022), this trend was not consistently observed in the intercropping systems analyzed. An increase in parasitoid abundance and parasitism rates was found when two or three crops were grown simultaneously, whereas reductions in herbivore populations occurred under all intercropping conditions (Fig. 6, Fig. S5). This may be because, in agricultural settings, plant species are often selected for specific functions (e.g., improving soil fertility, providing ground cover, or repelling pests). As a result, a well-designed system with fewer, strategically chosen species may equal or outperform a more diverse plant community. Push-pull systems illustrate this concept: they effectively manage pests and enhance parasitism services using just three species: a main crop, a pest-repellent crop, and a pest-attractive crop planted on the field’s perimeter (Cook et al. 2007).

Differences in sowing time between plant species in a field can lead to differences in plant biomass (Cotes et al. 2018). Plants with high biomass can more easily hide another, smaller crop plant from herbivores, and thus reduce abundance and damage. Our results partially support this premise, with reductions in herbivore abundance on the crop observed when the secondary crop is sown either before or at the same time as the main crop, but not after. However, contrary to expectations, a reduction in damage was only found when the secondary crop was sown after the main crop. We did not investigate whether it is more common to sow different types of intercrop at different times, so this could be an effect of specific crop combinations rather than the timing of sowing.

The use of companion plants (plants sown but not harvested) can be designed to provide services such as ground cover to reduce weed pressure but also to reduce herbivore pest abundance and damage (Lorin et al. 2015; Verret et al. 2017). However, the data available support the fact that herbivores and their damage are significantly reduced in systems with cash crops only, but not when companion crops are used. A significant increase in parasitoid abundance is also observed when only cash crops are used. A reason for this might be that companion plants are usually selected not to compete with the cash crop, and so potentially have less capacity to hide the cash crop from herbivores. Well-known examples of protective effects include ground-covering legumes as companion crops to oilseed rape, which are efficient in reducing herbivore pressure early in the season when plants are small, but their effect later in the season (when the oilseed rape is taller) is less clear (Seimandi-Corda et al. 2024; Magnin et al. 2025). In contrast, intercropped cash crops are more often of a similar stature, e.g., wheat and faba bean, barley and pea, and may therefore provide a stronger barrier effect.

#### Flower resources

Plant species diversity of flower margins, the size of the flower patches, the time since establishment of the patches, and the distance between the sampling location and the flowers can have an impact on arthropods. More diverse flower mixtures increase the complementarity of flower resource provision in space and time to better support beneficial arthropods (Scheper et al. 2013; Sutter et al. 2017). Pluriannual flower margins are less disturbed and provide better shelter for overwintering beneficials than annual margins. Larger flower patches provide more resources which support arthropod populations, and the populations of beneficial arthropods are expected to be higher in flower margins than in the crop. Consequently, it is expected to have more abundant beneficial populations close to flower patches than within the crop (Krimmer et al. 2019). Some of these effects are supported by Albrecht et al. (2020) for pollination services, but not for pest control. The present results show a more variable perspective. We found that parasitism rates increased significantly when flower resources were older than 1 year, but predator abundance increased significantly only when flower resources were younger than 1 year. Potentially, the soil disturbance involved in establishing flower resources in the first year, combined with the succession of the flower resource community over time, could favor different arthropod guilds at different times. Parasitism rates were also observed to be higher alongside less diverse flower resources (< 5 species) than in more diverse patches, and when samples were collected at least 1 m away from the flower resource into the crop. It is not clear what mechanisms could underlie these trends, so these results should be treated with caution until further research can shed more light on them. In particular, it is surprising that flower patch size had no effect on arthropods (Fig. 6, Fig. S5).

#### Semi-natural habitat (SNH)

It has been previously established that ecosystem service providers or pests can spill over from SNH to affect the crop (Rand et al. 2006; Boetzl et al. 2024). Our analysis indicates that such spillover effects may not always be positive, with an increase in herbivore abundance observed within 10 m of SNH. Some agricultural pests complete part of their development cycle in SNH, potentially explaining increased herbivore abundances near SNH (Juhel et al. 2017; Pigot et al. 2024). We also observed an increase in parasitism when woody SNH (hedgerows or woodland) was present, which may help to mitigate the effect of increased herbivore abundance (Fig. 6, Fig. S3). However, the data currently available on SNH remain limited, and additional studies are needed to confirm their effects. Landscape-level studies suggest that a higher proportion of grassland is associated with increased pollinator abundance, greater predation, or reduced pest pressure, while the proportions of forests and hedgerows appear to have weaker effects (Bartual et al. 2019; Perrot et al. 2023). To date, however, no meta-analysis has investigated how these different habitat types individually influence arthropods at the landscape scale.

#### Agroforestry

Few studies have tested the effect of agroforestry on arthropods, and thus, we have insufficient data to draw on to test the effects on predation, pollinators, and pollination. The only significant effect observed was the positive effect of alley cropping agroforestry on parasitoid abundance, which might reflect the fact that trees can provide refugia to beneficial arthropods and thus benefit their populations. However, not enough studies are currently available to test whether the benefit of agroforestry on ecosystem service providers is better supported by different configurations of tree plantations. In managed forests where coffee and cacao are grown, a positive relationship between the tree species diversity and the abundance of parasitoids and pollinators, and reduced pest infestation has been observed (Sperber et al. 2004; Nesper et al. 2017; Geeraert et al. 2019); but it is not sure if this relationship can be applied to alley cropping system.

### Limitations of the dataset and knowledge gaps

A key feature in our results is the high degree of between-study variability, leading to lower confidence in effects where less data is available. In some cases, it is therefore difficult to distinguish whether some practices or management factors truly have no effect or whether we do not have enough data to detect an effect. However, this variability itself is also informative, indicating that the effects are inconsistent. In that regard, our results support previous studies showing mixed effects of plant and habitat diversity on arthropods and their services (e.g., Karp et al. 2018). In separating the four diversification practices into different management strategies, we had hoped to explain some of this variation and to identify management strategies with more consistent effects than others. Although our results shed light on the effects of management practice (i.e., showing that all intercropping strategies reduce herbivores, while only some intercropping strategies increase parasitoids’ abundance), it does not seem that management is an overarching driver of the effect of plant diversification practices on arthropods. We initially sought to account for the context dependency of these effects by incorporating additional variables such as abiotic factors (e.g., climate) and more detailed information on plant and arthropod traits or phylogenetic groups. However, insufficient data prevented us from reliably testing these factors. Future studies that directly compare multiple management practices will be valuable for clarifying the efficacy of diversification strategies, but careful consideration of context will remain essential for interpreting their outcomes.

The high degree of between-study variability in our data meant that we tend to see more significant effects in categories for which we have more data. Notably, less data is available for agroforestry and maintenance of SNH than for intercropping and flower addition (Fig. S3). These smaller datasets likely contribute fewer significant results regarding these practices. Agroforestry and SNH are often considered practices that are challenging to implement, requiring a long establishment time and complex management, which may partially explain why they are less studied. They have also only recently gained substantial research attention (Fig. 3), while intercropping has been extensively studied, particularly for its agronomic benefits, for a much longer period. Flower resources have been recognized as a way to support biodiversity in agricultural landscapes (Jönsson et al. 2015), which may also explain why more studies are available here. However, another reason that we could not include many studies involving agroforestry and SNH is that they often compare plots along gradients of tree diversity or landscape coverage of SNH, which do not match our criteria for inclusion of the studies.

A lack of data is also observed for some types of responses, particularly for pollinator abundance and pollination service (Fig. 3). It highlights that even if crop diversification is a topic with an abundant literature (about 450 studies used in the present meta-analysis, and more studies that did not fit our criteria for inclusion), there is still a need for more research to be conducted. Interest in pollinators and their services is also relatively recent and is increasing with concerns about the negative impact of farming on populations of pollinators (Gemmill-Herren et al. 2021). It should also be noted that some service-providing arthropod groups, such as detritivores, are entirely missing from our study due to a lack of data. These organisms are important in nutrient cycles and for soil fertility, but are clearly understudied.

## Conclusions

Our study presents one of the largest and most up-to-date meta-analyses of the effect of plant diversification on arthropods and their services in agriculture. It shows that plant diversification has the potential to enhance beneficial arthropod populations and the ecosystem services they provide, while reducing herbivorous pest populations and their associated crop damage. By comparing diversification practices, we showed for the first time that the strongest and most consistent effects were observed for intercropping, leading to significant increases in predator and parasitoid populations, and reductions in herbivore abundance and damage. However, only limited effects were observed for the addition of flower resources, agroforestry, and SNH, as well as for certain key ecosystem services like pollination. These gaps highlight the need for further research.

Implementing plant diversification practices can require a substantial redesign of farming systems, or farmer cooperation and local governance in the case of SNH, that are not always owned by neighboring farmers. These changes must be supported by institutions, farmer networks, and evidence-based knowledge. Differences in the way diversification practices are implemented can lead to complex effects on arthropods, which are rarely taken into consideration in meta-analyses. An originality of the present study is to consider these management factors, showing that the effect of diversification practices is variable depending on how they are implemented and highly context-dependent. Consequently, while plant diversification shows clear potential to reduce pesticide reliance and support biodiversity, its practical implementation will require tailored strategies that account for local conditions. To promote the adoption of these practices, especially intercropping, policies that provide incentives and support for farmers are crucial. In the UK Sustainable Farming Incentive, intercropping is an option (https://www.gov.uk), but more advice as to effective combinations is necessary. While more research is needed to understand underexplored practices, there is sufficient evidence to justify action in favor of plant diversification, as overall these practices have the potential to pave the way to a more sustainable future food production.

## Supplementary information

Below is the link to the electronic supplementary material.

ESM 1 (DOCX 216 KB)

## Data Availability

Data used for the study can be found at https://zenodo.org/records/15023242.

## Code availability

R code used for the analysis is available in Supporting materials.
