# Supplementary material for: Implementation of practices shapes the effectiveness of agricultural diversification for arthropod related ecosystem services: a meta-analysis
Source: Agron Sustain Dev. 2026 Feb 26;46(2):19. doi: 10.1007/s13593-025-01082-7 (PMC12946007; doi:10.1007/s13593-025-01082-7)
Supplement: Supplementary file 1 — (DOCX 216 KB) [file 13593_2025_1082_MOESM1_ESM.docx]

**Supporting Information for**

Diversifying agriculture to enhance arthropod-driven ecosystem services: what kind of management matters?

Gaëtan Seimandi-Corda ^1,2*^, Chloe MacLaren ^1,3,4^, Kevin Tougeron ^5^, Johannes Forkman ^4^, Jess Hood ^1^, Andrew Mead ^1^, Amelia Dixon ^1^, Samantha M. Cook ^1^

^1^ Rothamsted Research, Harpenden, Hertfordshire, UK

^2^ Université de Toulouse, INRAE, UMR AGIR, Castanet-Tolosan F-31326, France

^3^ International Maize & Wheat Improvement Centre (CIMMYT), Southern Africa Regional Office (SARO), P.O. Box MP163, Harare, Zimbabwe

^4^ Department of Crop Production Ecology, Swedish University of Agricultural Sciences, Uppsala 75007, Sweden

^5^ Ecology of Interactions and Global Change Laboratory, Institute for Biosciences, Université de Mons, Mons, Belgium

Gaëtan Seimandi-Corda

**Email:**  gaetan.seimandi@outlook.fr

1. Literature search

The literature search, was conducted using Web of Science database (Core Collection) the first time on the 7/01/20 using the terms: (undersow* OR underseed* OR interseed* OR intercrop* OR “companion crop*” OR “companion plant*” OR “living mulch” OR interplant* OR “mixed crop” OR “flower strip*” OR “wildflower strip*” OR “flower margin” OR “field margin” OR "flower border" OR agroforestry OR "alley cropping" OR “trap crop*” OR hedgerow OR "field edge" OR "field boundaries" OR "crop diversification") AND (insect OR pollinat* OR herbivor* OR predat* OR parasit* OR biocontrol OR “biological control” OR “pest control” OR "natural enem*"). Were removed from the search the terms: nematod* OR pathogen* OR slug*. Only the research articles published in English were considered for this search. The first run of the search in Web of Science yielded 2,130 articles. This search was updated to include recently published articles on 14/01/21 (194 articles), 29/09/23 (890 articles), 11/06/24 (232 articles), making a total of 3,447 articles. Titles and abstracts of these articles were then checked and 991 articles dealing with crop diversification practices, including agroforestry, intercropping, addition of flower resources, and adjacent semi-natural habitats, and Arthropods were kept for further inspection (Fig. S1). In addition to these articles, 22 studies identified in other meta-analyses (Letourneau et al. 2011; Lichtenberg et al. 2017; Albrecht et al. 2020; Wan et al. 2020; Zamorano et al. 2020) were added. After a careful inspection of the content of the articles, 448 articles were kept for data extraction.

1. Data extraction

Data belonging to eight response categories were considered for extraction: the abundance of arthropod herbivores, predators, parasitoids, and pollinators, the plant damage level, the predation level, the parasitism level, the pollination level. For arthropod abundance, data such as the number of individuals per trap, per plant, or the percentage of plants infested were recorded. For the pollination service, measurements such as seed or fruit set were considered, while for the predation service, the number or percentage of prey items predated were extracted. For the parasitism service, the number or percentage of parasited hosts were recorded. Values related to the crop such as herbivory damage (damage recorded with scales, percentage of plants with damage, etc.) were also extracted. The mean or median values of the data presented for the control (low level of plant diversity) and treatments (high level of plant diversity) were extracted. If multiple data points were presented over a sampling season, values for each sampling occasion were collected. Data from illustrations were extracted using WebPlotDigitizer (Rohatgi 2015) with a three digits precision. For six articles (Dainese et al. 2017; Boetzl et al. 2019; Pecheur et al. 2020; McHugh et al. 2020; Staton et al. 2021; Bishop et al. 2023) data were retrieved from raw data available in Supporting Information. Mean values were computed directly from the data.

1. Data cleaning

After the data extraction the dataset was cleaned to remove redundant points. When multiple data types that were potentially redundant were extracted from the same study, only one was kept. We tried to keep the most informative and the most commonly used type of data in the dataset. For example, the abundance of arthropods per plant was preferred over the percentage of plants with arthropods.

When multiple data points were presented from two or more types of measurements belonging to one of the response categories, Pearson’s correlations between those values were checked. If the correlation was strong (*r* > 0.5) the most frequently observed type of measurement in the dataset was kept. In some publications, the same data were presented at different scales. This was the case when, for example, the abundance of a species was presented as an average value over the season and by the abundance on each sampling occasion. In this situation, the data points with the largest number of replicates were preferred if they allowed comparison with other response values when multiple types of data were collected. For example, if the abundance of an herbivore was presented both for the whole season and for each sampling occasion, but the abundance of predators presented only for each sampling occasion, the data collected for each occasion were preferred. Finally, redundant data points can be present if multiple taxonomic levels are presented. This was the case for example when the abundance of both the total number of predators and the abundance of different species of predators were presented in the same article. In this case, the higher taxonomic level was preferred to have higher abundance values. The preference for some types of data was subordinated to their quality. If redundant values were presented, the data points of higher quality were always preferred even if they did not respect the preferences presented above. The quality of the data was estimated by the quantity of information that can be related to the data points, such as the number of replicates, or the easiness by which the data can be retrieved from an illustration, and more generally of the quantity of information about the experiment (location, sampling occasion, treatment…) that can be retrieved from the data.

A total of 19,421 observations including treatment and control values were extracted from the 449 articles. The list of articles and the number of observations extracted per article is presented in the section “References used in the meta-analysis”.

1. Variable standardisation

For each observation several variables were recorded to be used as moderators in the statistical analysis. Some of the variables were initially recorded as numeric values (distance from habitat, etc.) but then transformed to categorical values to ensure the availability of a sufficient number of data points per category. When the information was not available in the article, a category “unspecified” was created to avoid the removal of the entire row when running models as it is normally the case when having non-attributed values (NAs). The variables considered are listed in Table S1.

1. Figures

Figure S1. PRISMA report based on (Page et al. 2021).


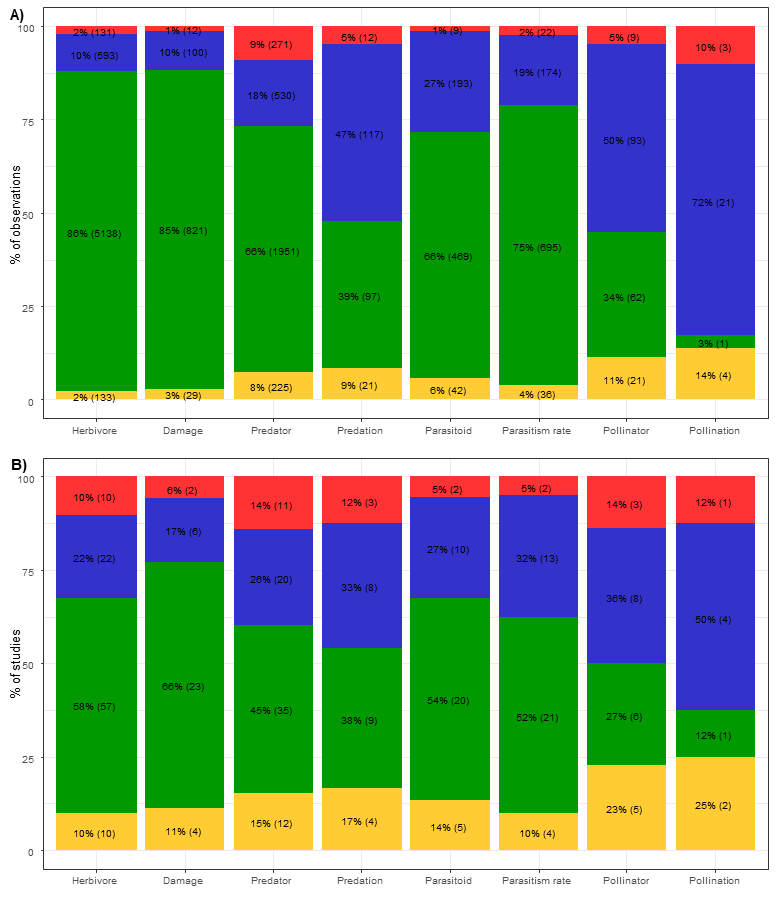


Figure S2. Distribution of the diversification practice categories used in the meta-analysis. A) Percentage (number) of observations used in the analysis per response type for different plant diversification practices. B) Percentage (number) of studies used in the analysis per response type for different plant diversification practices. red = agroforestry, blue = flower resource addition, green = intercropping, yellow = semi-natural habitats.

**Figure S3.** Percentage (number of observations / studies) used in the analysis per plant diversification practice for each category of management variables. a) agroforestry, b) semi-natural habitats (SNH), c) flower resource addition, and d) intercropping.


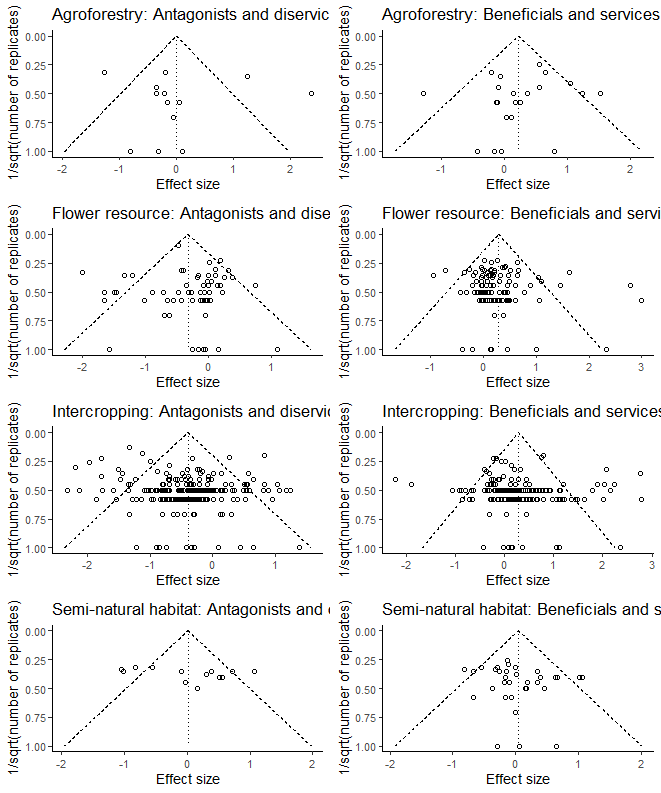


**Figure S4:** Funnel plots showing the mean effect size within each study in relation to the inverse square root of the number of replicates in each study. Data are grouped into tests of effects on ‘antagonists and disservices’ (left column) and on ‘beneficials and services’ (right column). The dashed vertical lines indicate the mean effect size across all studies within each group, while the ‘funnels’ indicate an expectation of increasing precision in effect size estimates as the number of replicates increases (these are calculated as the effect size plus or minus one over the square root of the number of replicates, multiplied by 1.96). Asymmetry in the spread of points on the funnel plots, particularly in the lower part of the plot, can indicate publication bias. We do not see any cause for concern in these plots, with points in the lower parts of each plot typically spread evenly to both left and right of the centre. Possibly, there are a few missing studies for beneficials and antagonists with intercropping and flower resources showing a lower than expected or negative effect size (points with few replicates spread further to the right). However, given most studies in our dataset are further up the plots with more replicates, and where many points are spread to both sides, we do not expect this hint of publication bias to have strongly influenced our results.

NB: two outliers with large effect sizes are not shown (one for flower resources – antagonists and disservices, and the other for; these were caused by studies reporting zero values that were replaced by 0.0001 for log transformation, and thus resulted in apparently large effect sizes. Given the arm-based model does not rely on within-study effect sizes, these points would not have influenced our analyses.


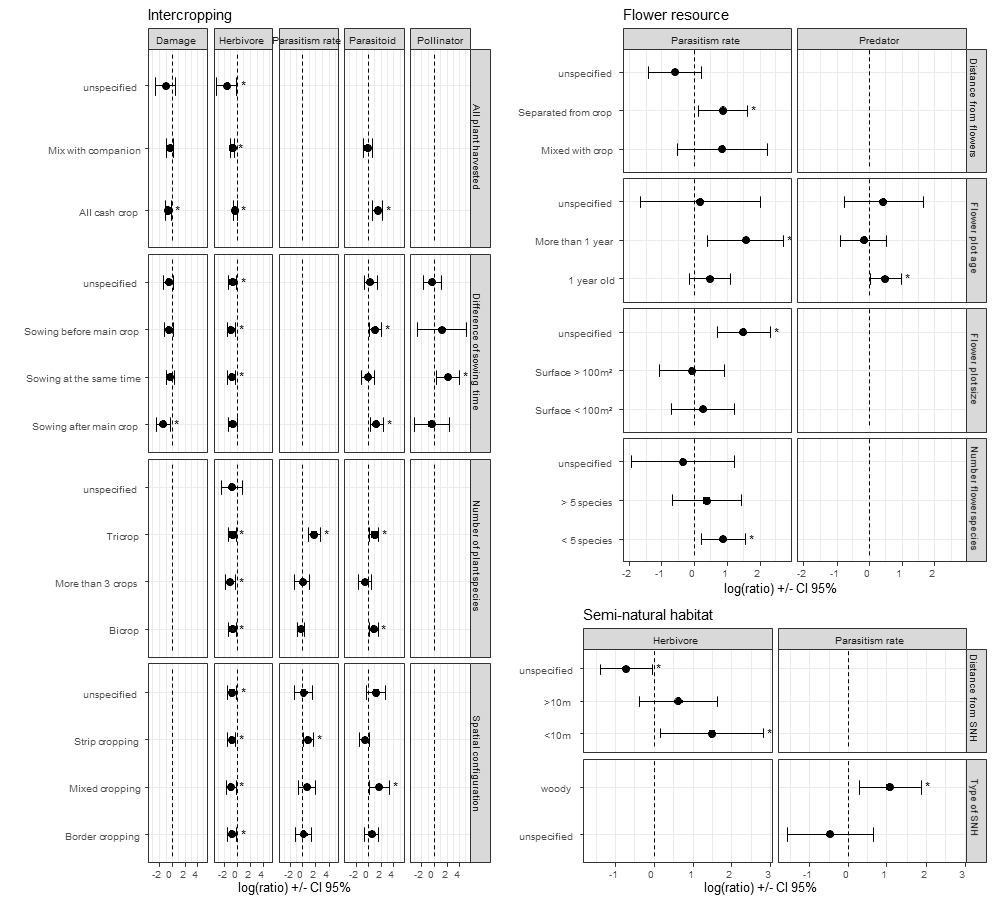


**Figure S5.** Log of ratio (± CI 95 %) of the effect of plant diversification practices on different response types depending on the management variables tested in three diversification practices. No significant effects of variables are observed for the management variables related to agroforestry and consequently are not represented. * denotes significant differences between control and diversified treatments.

1. **Tables**

Table S1. List of the variables collected during the data extraction

| **Variables** | **Diversification practice related** | **Description** |
| --- | --- | --- |
| Type of diversification practice |  | Four practices were considered: |
|  |  | 1) Agroforestry includes any crop (perennial or annual) grown in the vicinity of perennial woody plants and includes cropping systems in alley cropping or shaded forest of cacao plantation (see below). |
|  |  | 2) Flower resources addition includes any condition where the main purpose of the diversification practice is to deliver nectar or pollen to ecosystem service providers. Most often this practice is applied with flower margins or strips, but flowers can also be mixed spatially within the crop. |
|  |  | 3) Intercropping considers all types of plant association where the main purpose of the additional plant species is not to bring flower resources and do not fit in the agroforestry criteria. This includes for example, but not only, trap cropping, relay cropping, strip cropping, pixel planting. |
|  |  | 4) Semi-natural habitat (SNH) considered experiments with crops alongside hedgerows, forests, grasslands or grassy strips. |
| Distance between the flowers or SNH and the sampling point | Flower resources and semi-natural habitats | This variable is categorised as mixed within the crop (1 m or less) or separated (more than 1 m away from the crop). |
| Number of wildflower species | Flower resources | Categorised as equal or less than 5 species, and greater than 5 species. |
| Size of the flower plot | Flower resources | Categorised as less than 100 m² and greater than 100 m² |
| Age of the flower plot | Flower resources | Categorised as 1 year or older. |
| Type of semi-natural habitats | Semi-natural habitats | Categorised as woody (forest, hedgerows) or herbaceous (grassland, grassy strip). |
| Type of trees in agroforestry | Agroforestry | Categorised as fruit and nut trees, or diverse (wild and timber trees). |
| Spatial configuration of agroforestry | Agroforestry | Categorised as alley cropping (lines of trees interplanted with crops), or managed forest (crops growing under the canopy of a forest receiving low maintenance by humans). |
| Type of crops | Intercropping | Plot having only cash-crops (plants that are harvested and commercialised) or association with a companion plant (plant providing a service but not used for commercial purpose). |
| Spatial configuration of intercropping | Intercropping | The spatial configuration is categorised between: |
|  |  | 1) border cropping, also called perimeter cropping, which is often observed for trap-crop systems, |
|  |  | 2) mixed cropping when plant species are sown without specific spatial pattern or alternated at a very fine scale (pixel cropping), |
|  |  | 3) and strip cropping including systems with different strips allowing mechanical harvest or alternate rows of different species. |
| Number of plant species | Intercropping | The number of plant species grown simultaneously categorised as bicrop, tricrop and more than 3 crops. |
| Sowing time | Intercropping | If the secondary crops are sown or planted before, at the same time or after the main crop. |

**Table S2.** Results of the ANOVAs performed on the models (see Fig. 1) used in the meta-analysis. Bold cells represent significant results.

| **Response aggregation** | **Beneficial and services / Antagonists and disservices** | | **Separated response types** | | | | | |
| --- | --- | --- | --- | --- | --- | --- | --- | --- |
| **Practices aggregation** | **All practices together** | **Separated practices** | **Separated practices** | **Agroforestry** | **Flower resource** | **Intercropping** | **Semi-natural habitat** |  |
|  |  |  |  |  |  |  |  |  |
| Variables | Model 1 | Model 2 | Model 3 | Model 4 | Model 5 | Model 6 | Model 7 |  |
| Response type | **χ² = 28, df = 1, p < 0.001** | **χ² = 28.7, df = 1, p < 0.001** | **χ² = 41.4, df = 7, p < 0.001** | χ² = 5 df = 6, p = 0.547 | **χ² = 30.35, df = 7, p < 0.001** | **χ² = 28.87, df = 7, p < 0.001** | **χ² = 22.48, df = 7, p = 0.002** |  |
| Diversification practice | χ² = 0.8, df = 1, p = 0.371 | χ² = 9.2, df = 4, p = 0.057 | **χ² = 13, df = 4, p = 0.011** |  |  |  |  |  |
| Study | **χ² = 833.3, df = 488, p < 0.001** | **χ² = 810.4, df = 448, p < 0.001** | **χ² = 917.1, df = 448, p < 0.001** | **χ² = 71.6, df = 26, p < 0.001** | **χ² = 150.3 df = 105, p = 0.003** | **χ² = 680.3 df = 287, p < 0.001** | **χ² = 91.76, df = 35, p < 0.001** |  |
| Response type: Diversification practice | **χ² = 105.6, df = 1, p < 0.001** | **χ² = 94.8, df = 4, p < 0.001** | **χ² = 138.7, df = 28, p < 0.001** | χ² = 8.6, df = 7, p = 0.284 | **χ² = 20.4, df = 8, p < 0.001** | **χ² = 67.7, df = 8, p < 0.001** | χ² = 14.26, df = 8, p = 0.075 |  |
| Response type: Diversification practice : Agroforestry configuration |  |  |  | χ² = 3.05, df = 6, p = 0.803 |  |  |  |  |
| Response type: Diversification practice : Type of trees : Study |  |  |  | **χ² = 11.1, df = 4, p = 0.025** |  |  |  |  |
| Response type: Diversification practice : Distance from flowers : Study |  |  |  |  | χ² = 23.5, df = 16, p = 0.1 |  |  |  |
| Response type: Diversification practice : Number of flowers: Study |  |  |  |  | χ² = 6.59, df = 16, p = 0.98 |  |  |  |
| Response type: Diversification practice : Patch size : Study |  |  |  |  | χ² = 15.6, df = 16, p = 0.484 |  |  |  |
| Response type: Diversification practice : Duration of flower : Study |  |  |  |  | χ² = 12.39, df = 14, p = 0.575 |  |  |  |
| Response type: Diversification practice : Type of intercropping : Study |  |  |  |  |  | **χ² = 36.23, df = 10, p < 0.001** |  |  |
| Response type: Diversification practice : Intercropping configuration : Study |  |  |  |  |  | χ² = 24.8, df = 19, p = 0.167 |  |  |
| Response type: Diversification practice : Number of species : Study |  |  |  |  |  | **χ² = 47.16, df = 16, p < 0.001** |  |  |
| Response type: Diversification practice : Sowing timing : Study |  |  |  |  |  | **χ² = 33.78, df = 20, p = 0.028** |  |  |
| Response type: Diversification practice : distance to SNH : Study |  |  |  |  |  |  | **χ² = 40, df = 11, p < 0.001** |  |
| Response type: Diversification practice : Type of SNH : Study |  |  |  |  |  |  | χ² = 11, df = 9, p = 0.274 |  |

**Table S3.** Summary of the effect of different management factors in studies using selected plant diversification practices compared to monocrops on different response categories. Red arrows indicate a decrease of the response for a level of the variable tested, a green arrow indicates an increase, and a bared zero (ø) indicates that not enough points were available to test this effect. Coloured cells (red or green) show significant results while the number associated represents the number of observations of the diversified treatment (and number of studies) available. SNH: Semi-Natural Habitats.

|  |  | **Herbivore** | **Damage** | **Predator** | **Predation** | **Parasitoid** | **Parasitism** | **Pollinator** | **Pollination** |
| --- | --- | --- | --- | --- | --- | --- | --- | --- | --- |
| Agroforestry | Alley cropping | **↗ 112 (9)** | **↘ 12 (2)** | **↗ 253 (14)** | **Ø 6 (1)** | **↗ 9 (2)** | **↘ 20 (1)** | **Ø 2 (2)** | **Ø 0** |
|  | Managed forest | **↘ 19 (4)** | **Ø 0** | **↗ 18 (2)** | **Ø 6 (2)** | **Ø 0** | **↘ 2 (1)** | **Ø 2 (1)** | **Ø 0** |
|  | Fruits and nuts | **↗ 50 (7)** | **↘ 2 (1)** | **↗ 95 (9)** | **Ø 6 (1)** | **↗ 8 (1)** | **Ø 0** | **Ø 0** | **Ø 0** |
|  | Diverse tree functions | **↘ 81 (6)** | **↗ 10 (1)** | **↗ 176 (7)** | **Ø 6 (2)** | **↗ 1 (1)** | **↘ 22 (2)** | **Ø 4 (3)** | **Ø 0** |
| Intercropping | Cash crops | **↘ 3174 (141)** | **↘ 566 (42)** | **↗ 1252 (73)** | **↘ 6 (3)** | **↗ 316 (30)** | **↗ 550 (37)** | **↗ 25 (6)** | **↘ 1 (1)** |
|  | Companion crops | **↘ 1844 (94)** | **↘ 218 (29)** | **↗ 641 (53)** | **↗ 91 (9)** | **↘ 153 (15)** | **↗ 145 (15)** | **↗ 37 (5)** | **Ø 0** |
|  | Strip cropping | **↘ 3404 (143)** | **↘ 580 (43)** | **↗ 1428 (85)** | **↘ 64 (8)** | **↗ 381 (32)** | **↘ 555 (37)** | **↗ 37 (5)** | **Ø 0** |
|  | Border cropping | **↘ 732 (44)** | **↘ 91 (17)** | **↗ 237 (17)** | **↘ 7 (1)** | **↗ 30 (8)** | **↗ 38 (5)** | **Ø 0** | **Ø 0** |
|  | Mixed cropping | **↘ 649 (29)** | **↘ 77 (10)** | **↗ 149 (16)** | **↗ 26 (2)** | **↗ 30 (3)** | **↗ 69 (5)** | **↗ 9 (3)** | **Ø 0** |
|  | 2 crops | **↘ 4464 (204)** | **↘ 738 (64)** | **↗ 1748 (107)** | **↘ 63 (9)** | **↗ 413 (40)** | **↘ 660 (44)** | **↗ 50 (9)** | **↘ 1 (1)** |
|  | 3 crops | **↘ 481 (37)** | **↘ 61 (17)** | **↗ 125 (21)** | **↘ 32 (2)** | **↗ 48 (10)** | **↗ 27 (8)** | **↗ 6 (2)** | **Ø 0** |
|  | >3 crops | **↘ 176 (18)** | **↘ 22 (6)** | **↘ 63 (12)** | **↘ 2 (2)** | **↘ 8 (4)** | **↘ 8 (2)** | **↗ 6 (1)** | **Ø 0** |
|  | Sowing before | **↘ 1926 (66)** | **↘ 199 (18)** | **↗ 621 (33)** | **↗ 87 (7)** | **↗ 122 (11)** | **↘ 222 (11)** | **↗ 2 (1)** | **Ø 0** |
|  | Sowing simultaneously | **↘ 1678 (87)** | **↘ 370 (32)** | **↗ 591 (38)** | **↘ 4 (2)** | **↘ 88 (9)** | **↗ 330 (17)** | **↗ 18 (4)** | **Ø 0** |
|  | Sowing after | **↘ 616 (34)** | **↘ 87 (8)** | **↗ 229 (23)** | **Ø 0** | **↗ 148 (15)** | **↗ 64 (6)** | **↘ 29 (3)** | **Ø 0** |
| Flower resource | Separated from crops | **↘ 121 (15)** | **↘ 22 (5)** | **↗ 131 (14)** | **↗ 39 (7)** | **↘ 13 (6)** | **↗ 54 (12)** | **↘ 11 (3)** | **↘ 3 (1)** |
|  | Mixed with crops | **↘ 111 (11)** | **↘ 6 (2)** | **↗ 72 (9)** | **↗ 37 (6)** | **↗ 27 (4)** | **↗ 27 (5)** | **↘ 24 (5)** | **↗ 3 (2)** |
|  | < 5 species | **↘ 365 (25)** | **Ø 34 (5)** | **↗ 327 (29)** | **↗ 71 (9)** | **↗ 175 (14)** | **↗ 120 (20)** | **↗ 15 (7)** | **↗ 5 (3)** |
|  | > 5 species | **↘ 144 (17)** | **Ø 38 (5)** | **↗ 155 (21)** | **↗ 31 (11)** | **↗ 9 (5)** | **↗ 44 (8)** | **↗ 73 (15)** | **↗ 14 (6)** |
|  | Surface < 100m² | **↗ 346 (17)** | **Ø 64 (5)** | **↗ 222 (18)** | **↗ 31 (8)** | **↗ 154 (9)** | **↗ 62 (11)** | **↘ 22 (6)** | **Ø 2 (1)** |
|  | Surface > 100m² | **↘ 94 (11)** | **Ø 2 (1)** | **↗ 148 (14)** | **↗ 38 (4)** | **↗ 16 (5)** | **↘ 38 (8)** | **↗ 45 (12)** | **Ø 12 (5)** |
|  | 1 year old | **↘ 448 (40)** | **Ø 70 (8)** | **↗ 411 (44)** | **↗ 70 (14)** | **↗ 161 (18)** | **↗ 153 (28)** | **↘ 39 (14)** | **Ø 6 (3)** |
|  | >1 year old | **↘ 105 (14)** | **Ø 12 (2)** | **↘ 89 (11)** | **↗ 45 (8)** | **↗ 23 (5)** | **↗ 11 (3)** | **↘ 45(8)** | **Ø 8 (3)** |
| SNH | < 10m from SNH | **↗ 3 (1)** | **↗ 2 (1)** | **↗ 6 (2)** | **Ø 0** | **↗ 3 (2)** | **Ø 0** | **Ø 0** | **Ø 0** |
|  | > 10m from SNH | **↗ 53 (3)** | **↗ 14 (2)** | **↗ 54 (10)** | **Ø 4 (1)** | **↗ 29 (4)** | **↗ 29 (3)** | **↘ 8 (2)** | **Ø 0** |
|  | Woody habitat | **↗ 65 (7)** | **↘ 1 (1)** | **↗ 149 (16)** | **Ø 17 (4)** | **↗ 32 (3)** | **↗ 31 (4)** | **↘ 19 (7)** | **↗ 4 (2)** |
|  | Herbaceous habitat | **↗ 7 (2)** | **↗ 2 (1)** | **↗ 32 (5)** | **Ø 0** | **Ø 0** | **Ø 0** | **Ø 0** | **Ø 0** |

**Supporting information references:**

Albrecht M, Kleijn D, Williams NM, et al (2020) The effectiveness of flower strips and hedgerows on pest control, pollination services and crop yield: a quantitative synthesis. Ecology Letters 23:1488–1498. https://doi.org/10.1111/ele.13576

Bishop GA, Fijen TPM, Desposato BN, et al (2023) Hedgerows have contrasting effects on pollinators and natural enemies and limited spillover effects on apple production. Agriculture, Ecosystems & Environment 346:108364. https://doi.org/10.1016/j.agee.2023.108364

Boetzl FA, Krimmer E, Krauss J, Steffan‐Dewenter I (2019) Agri‐environmental schemes promote ground‐dwelling predators in adjacent oilseed rape fields: Diversity, species traits and distance‐decay functions. Journal of Applied Ecology 56:10–20. https://doi.org/10.1111/1365-2664.13162

Dainese M, Montecchiari S, Sitzia T, et al (2017) High cover of hedgerows in the landscape supports multiple ecosystem services in M editerranean cereal fields. Journal of Applied Ecology 54:380–388. https://doi.org/10.1111/1365-2664.12747

Letourneau DK, Armbrecht I, Rivera BS, et al (2011) Does plant diversity benefit agroecosystems? A synthetic review. Ecol Appl 21:9–21. https://doi.org/10.1890/09-2026.1

Lichtenberg EM, Kennedy CM, Kremen C, et al (2017) A global synthesis of the effects of diversified farming systems on arthropod diversity within fields and across agricultural landscapes. Global Change Biology 23:4946–4957. https://doi.org/10.1111/gcb.13714

McHugh NM, Moreby S, Lof ME, et al (2020) The contribution of semi‐natural habitats to biological control is dependent on sentinel prey type. Journal of Applied Ecology 57:914–925. https://doi.org/10.1111/1365-2664.13596

Page MJ, McKenzie JE, Bossuyt PM, et al (2021) The PRISMA 2020 statement: an updated guideline for reporting systematic reviews. BMJ 372:n71. https://doi.org/10.1136/bmj.n71

Pecheur E, Piqueray J, Monty A, et al (2020) The influence of ecological infrastructures adjacent to crops on their carabid assemblages in intensive agroecosystems. PeerJ 8:e8094. https://doi.org/10.7717/peerj.8094

Rohatgi A (2015) WebPlotDigitizer Version 3.9. Retrieved September 22:2015

Staton T, Walters RJ, Smith J, et al (2021) Evaluating a trait‐based approach to compare natural enemy and pest communities in agroforestry vs. arable systems. Ecological Applications 31:e02294. https://doi.org/10.1002/eap.2294

Wan N-F, Zheng X-R, Fu L-W, et al (2020) Global synthesis of effects of plant species diversity on trophic groups and interactions. Nat Plants 6:503–510. https://doi.org/10.1038/s41477-020-0654-y

Zamorano J, Bartomeus I, Grez AA, Garibaldi LA (2020) Field margin floral enhancements increase pollinator diversity at the field edge but show no consistent spillover into the crop field: a meta‐analysis. Insect Conserv Diversity 13:519–531. https://doi.org/10.1111/icad.12454
